# Visual hallucinosis and Linezolid use: A case report.

**DOI:** 10.1192/j.eurpsy.2023.2149

**Published:** 2023-07-19

**Authors:** M. Fernández Fariña, C. E. Regueiro Martín-Albo, M. E. Expósito Durán, F. Mayor Sanabria, Í. Alberdi Páramo

**Affiliations:** Instituto de Psiquiatria y Salud Mental, Hospital Clínico San Carlos, Madrid, Spain

## Abstract

**Introduction:**

We present the case of a 78-year-old man with multiple somatic pathologies and associated depressive symptoms, under treatment with Citalopram 10mg, who was admitted due to cholangitis secondary to biliary prosthetic obstruction.

Empirical antibiotic treatment with Meropenem and Linezolid was started, along with an increase in the dose of Citalopram to 20mg due to mood worsening. The patient begins with symptoms consisting of complex and polymorphic visual hallucinosis, without any affective or behavioral repercussions. He does not present another semiology of the psychotic sphere.

**Objectives:**

To highlight the importance of knowing the different interactions and adverse effects of drugs for good clinical management.

**Methods:**

We collected the complete medical history of our patient and we carried out a review of the interactions and adverse effects described with the antibiotic drug Linezolid.

**Results:**

As the onset of hallucinations was temporarily correlated with the use of medications, drug-induced hallucinations were suspected, resolving completely after 2 days after withdrawal of Linezolid treatment.

Linezolid is a nonselective inhibitor of MAO A and B, preventing the destruction of monoamine neurotransmitters like serotonin, dopamine, or norepinephrine. It has dopaminergic properties that may enhance the central nervous system effects of anticholinergics and co-prescription with serotonergic drugs increases the risk of serotonin syndrome.

**Conclusions:**

This case highlights the importance of taking into account drug interactions and adverse effects to reduce the risk of drug induced symptoms and optimize their management.

The increase in resistance to antibiotic treatment allows us to anticipate that the use of Linezolid will increase in the coming years, and it is important to know its mechanism of action given the interactions with psychotropic drugs that we use in our usual clinical practice

**Disclosure of Interest:**

None Declared

